# Keeping one's bursts under control: A protease/inhibitor switch regulates reactive oxygen species production during immunity

**DOI:** 10.1093/plcell/koad264

**Published:** 2023-10-11

**Authors:** Mariana Schuster

**Affiliations:** Assistant Features Editor, The Plant Cell, American Society of Plant Biologists; Leibnitz Institute of Plant Biochemistry, 06108, Halle (Saale), Germany

Plants can detect and respond to pathogen attack due to their remarkable immune system. The plant immune system is now understood as a pathway that integrates 2 branches (Yuan et al. 2021). In the first branch, termed Pattern-triggered immunity (PTI), basal defenses that repel most pathogens are initiated when membrane receptors perceive molecules from microorganisms. In the second branch of Effector-triggered immunity, strong, specific, and localized defenses are deployed against discrete groups of pathogens that manage to circumvent PTI. In this case, molecules of these pathogens are perceived either inside the cell or at the plasma membrane.

One of the main defense responses during PTI is the rapid and transient production (burst) of reactive oxygen species (ROS) in the space between cells. ROS serve a dual role during immunity that depends on their accumulation level. Low levels of ROS are associated with signal transduction, whereas high levels cause oxidative damage to the pathogen (Wu et al. 2023). RBOH protein D (RBOHD) is the principal ROS-producing enzyme during PTI in *Arabidopsis thaliana*. RBOHD accumulation and function are regulated via phosphorylation and ubiquitination, and the protein is degraded in the vacuole by an unknown enzyme (Lee et al. 2020). In this issue, **Yang Liu and colleagues** (Liu et al. 2023) report that the papain-like cysteine protease Xylem cysteine peptidase 1 (XCP1) is the protease that destabilizes RBOHD in the vacuole. Moreover, by studying XCP1 inhibitor cystatin 6, they uncover a new mechanism of regulation of RBOHD stability.

Cystatins are protease inhibitors and regulators of plant immunity (Lima et al. 2015). To study the Arabidopsis cystatins that act in PTI, Liu and colleagues challenged cystatin mutants of Arabidopsis with the bacterial pathogen *Pseudomonas syringae* pv. *maculicola* ES4326 (*Psm* ES4326) and identified *cys6* as a mutant with impaired basal defenses. To investigate if CYS6 regulates PTI, the authors infiltrated *cys6* plants with the PTI-inducing peptide Elf18 and measured PTI responses including the ROS burst and MPK3/6 phosphorylation. PTI responses were abolished in the *cys6* plants, suggesting that CYS6 regulates PTI. Furthermore, the authors concluded that CYS6 function in regulating PTI is dependent on its protease inhibitor function by using a transgenic line expressing a mutant of CYS6 unable to inhibit proteases.

Liu et al. used split-luciferase complementation and pull-down assays to identify XCP1 as the protease inhibited by CYS6 in the vacuole. *xcp1* mutants showed enhanced PTI responses when infiltrated with Elf18, suggesting XCP1-mediated negative regulation of PTI. Given that RBOHD is degraded in the vacuole where XCP1 resides, Liu et al. enquired whether XCP1 destabilizes RBOHD in this compartment. They indeed measured higher RBOHD protein levels in *xcp1* Arabidopsis lines compared to the control. Furthermore, the coexpression of AtRBOHD and AtXCP1 or AtXCP1^C161A^ (catalytically dead mutant) in *Nicotiana benthamiana* followed by measurement of AtRBOHD protein levels, showed that XCP1 is the enzyme that degrades RBOHD in the vacuole (Fig. 1). Finally, the addition of AtCYS6 inhibited XCP1 function and therefore RBOHD degradation.

**Figure 1.**
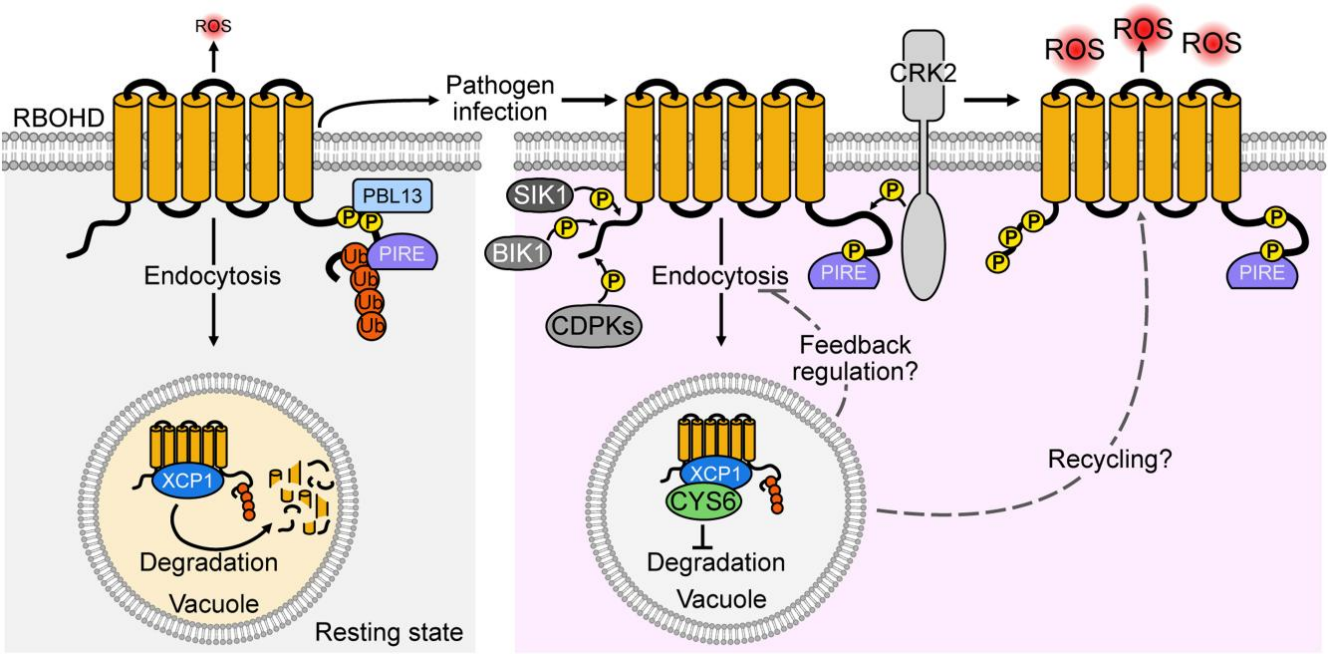
Regulation of the stability of RBOHD upon pathogen invasion. RBOHD is degraded in the vacuole by XCP1. Upon pathogen infection, RBOHD function and stability are regulated not only via phosphorylation but also by the inhibition of XCP1-mediated degradation via CYS6. Reprinted from Liu et al. (2023), Figure 10.

RBOHD accumulates during PTI. By analyzing RBOHD protein levels in the *cys6* mutant during PTI, Liu et al. showed that this accumulation is dependent on CYS6 function. The report of Liu et al. expands our knowledge of the regulation of ROS burst during immunity by describing the XCP1-CYS6 protease inhibitor switch of RBOHD accumulation.

## Dive Curated Terms

The following phenotypic, genotypic, and functional terms are of significance to the work described in this paper:

XCP1 Gramene: AT4G35350
XCP1 Araport: AT4G35350

RBOHD Gramene: At5g47910
RBOHD Araport: At5g47910

PTI Gramene: pattern-triggered immunity
PTI Araport: pattern-triggered immunity
